# Antipsychotic-induced weight gain: exploring the role of psychiatrists in managing patients’ physical health – challenges, current options and direction for future care

**DOI:** 10.1192/bjb.2023.29

**Published:** 2024-02

**Authors:** Kenn Lee, Adeola Akinola, Seri Abraham

**Affiliations:** 1 Pennine Care NHS Foundation Trust

**Keywords:** Antipsychotics, ethics, obesity, schizophrenia, medical negligence

## Abstract

Antipsychotics and severe mental illness (SMI) are associated with weight gain, and obesity increases the risks of cardiometabolic disease and premature death. These present management and liability issues for psychiatrists. Physical healthcare for people with SMI is poor, and this may partly be owing to training limitations and lack of proactiveness by psychiatrists. Ethically and legally, psychiatrists have a duty to avoid unnecessary harm and to maintain an acceptable standard of care. This would apply particularly to patients receiving compulsory treatment for their SMI owing to their vulnerability. Discrepancy between psychiatric and non-psychiatric approaches to pharmacological treatment creates ambiguity, and weight gain could demotivate antipsychotic adherence. This article explores how the Mental Health Act could be used to address these issues, and the ethical considerations, and proposes how long-acting glucagon-like peptide-1 receptor agonists could be introduced into existing psychiatric practice as a treatment option for antipsychotic-induced weight gain and obesity in SMI.

Antipsychotics are a class of medications regularly used in psychiatry. They are indicated in a variety of severe mental illnesses (SMIs) including schizophrenia, bipolar affective disorder and depression. They are also increasingly prescribed during pregnancy^1,2^ and in children^3,4^ and individuals with learning difficulty,^5,6^ often off-label.

They are also associated with weight gain and other cardiometabolic side-effects.^7,8^ This is a potential problem as excess weight is more prevalent in people with SMI, with 25.9% being categorised as obese and 60.1% as either obese or overweight,^9^ compared with 13% and 39%, respectively, in the general population.^10^ According to the World Health Organization, obesity is the fifth leading risk factor for death worldwide^11^ and contributes to a wide range of long-term health complications, including type 2 diabetes mellitus (T2DM), heart disease and musculoskeletal conditions such as arthritis.^10^ Consequently, it stands to reason that antipsychotic-induced weight gain (AIWG) may contribute to poorer physical health, premature death and the 10- to 20-year mortality gap seen in people with SMI.^12^ This is especially because antipsychotics are often prescribed long-term – if not indefinitely – and the harm from their cardiometabolic effects accumulate over time, with patients progressively gaining weight the longer they are on the medication.^13^

This is further complicated when compulsory treatment is involved. Unlike in most areas of medicine, SMI patients may not always be fully consenting to psychiatric treatment. The precise limits and frameworks will differ by country and territory. In England and Wales, this is mainly through sections IV and 4A of the Mental Health Act 1983 (MHA1983), which outline the powers and limits psychiatrists have when treating patients committed into their care.

These laws exist owing to the nature of many SMIs, such as schizophrenia and mania, where patients can lose insight and/or capacity when acutely unwell. Instead, treatment may be initiated by the psychiatrist during this period, with the medication choice made by them rather than the patient. In the case of psychoses, this most commonly takes the form of second-generation antipsychotics, which are particularly prone to causing AIWG and other cardiometabolic effects. Once treated, patients are then faced with the difficult choice of whether to remain on the obesogenic medication, discontinue treatment or switch to a different medication. The latter two options carry a risk of becoming mentally unwell again, at which point the offending medication may once again be reinitiated.

## Practice challenges

### Quality of care

When clinicians make treatment decisions on behalf of their patients, those decisions are expected to be ones that are in the patient's best interests. It therefore stands to reason that greater care would be expected of clinicians to minimise iatrogenic harm caused by compulsory treatment compared with fully consensual treatment. This is because patients lack the option of withdrawing consent and discontinuing compulsory treatment as a means of mitigating unwanted effects. In the case of antipsychotic use by psychiatrists to treat SMI, this would include monitoring for and minimising the risk of developing AIWG and other cardiometabolic effects.

Yet, physical health monitoring and management are generally poor for patients with SMI.^7^ Physical examination skills among psychiatrists were found to worsen with seniority in a national survey,^14^ limiting training and learning opportunities through seniors. Moreover, concern over cardiometabolic effects and recognition of the role of mental health professionals in physical health monitoring for SMI patients have not always translated into clinical practice, with the issue having long been recognised yet continuing to recur across subspecialties and borders.^15–19^ In the case of obesity, this has previously been attributed to lack of training and confidence around management.^20^ There may also be a lack of proactiveness from healthcare professionals, with the condition going unrecognised in patients’ clinical notes, clinicians underestimating the severity of patients’ excess weight, clinicians lacking awareness of treatment options and having a negative attitude towards their efficacy, and the perception among clinicians that obesity arises from lifestyle choices with weight management being primarily the responsibility of the patient.^11,21^

### Current standard practice

Multiple guidelines have been published around managing obesity, for the general population^22–25^ and specific to AIWG and SMI.^7,26,27^ They broadly align in terms of management, taking a stepwise approach beginning with conservative interventions (e.g. diet, exercise, behaviour interventions). They then recommend escalating to pharmacological therapy (e.g. switching from or reducing medications that cause weight gain, adding a weight-loss medication (WLM)) and then to bariatric surgery if the previous step is ineffective.

However, there is a divide in the approaches taken by psychiatric and non-psychiatric guidelines around WLM choice. Guidelines produced by national bodies, endocrinology and cardiology consistently recommend using only licensed WLMs for pharmacological treatment of obesity. This sits alongside a well-researched body of literature on the comparative efficacy of these treatments.^28,29^ By comparison, the efficacy of licensed WLMs in AIWG is less well researched,^30^ with guidelines by psychiatric and psychopharmacological bodies instead recommending off-label treatments, particularly off-label use of metformin.^7,27^ This is despite explicit recommendations against its use by endocrinologists.^24,25^ Current psychiatric guidelines are in need of updating, as they have yet to incorporate recent developments – such as new candidate medications (e.g. olanzapine/samidorphan)^31^ or recently published human trials^30^ – and continue to include medications withdrawn from the market (e.g. lorcaserin).

### Maintaining adherence

It is recognised that non-adherence is an issue in chronic illness and a particular challenge in schizophrenia.^32^ This includes non-adherence to prescribed medication, with 24.3% to 52.2% of those prescribed oral antipsychotic having unsatisfactory adherence,^33^ as well as to ‘healthy’ lifestyle changes such as diet and exercise.^32^ This is in spite of adherence-promoting interventions such as electronic reminders, long-acting injections (‘depot’ medication), psychoeducation and compulsory treatment.

The reasons can be complex, including – among others – illness-specific, medication-specific and environmental factors. In addition to insight loss, patients with schizophrenia may experience negative symptoms, cognitive dysfunction and social isolation.^32^ They are likelier to reside in more deprived areas, occupy lower socioeconomic positions and face greater marginalisation, limiting their access to health-related resources such as medical services, exercise opportunities and healthy food options.^34,35^ These factors make it more difficult for patients to adhere to treatment.

When looking at antipsychotics specifically, the prevalence of side-effects is high, with >75% of those prescribed antipsychotics experiencing one or more side-effects and 34.7% developing weight gain specifically.^36^ In addition to the direct health consequences and objective severity of side-effects, patients’ subjective experience and personal values may cause certain side-effects to be less tolerable. Excessive and sudden weight gain affect one's self-image, self-esteem and quality of life.^37^ This could be particularly distressing to patients already having to come to terms with having an SMI diagnosis and the stigma associated with it. Weight gain is outwardly visible and hard to conceal, potentially making it particularly undesirable. Consequently, such side-effects may further lower patients’ motivation to adhere to treatment, especially when they are symptom-free with respect to the underlying SMI.

### Ethical considerations

A common adage for healthcare professionals is to ‘first, do no harm’. Non-maleficence is a principle in medical ethics that calls for clinicians to avoid unnecessary harm to patients, be it by action or inaction.^38^ To this end, they should explain to patients the potential unwanted effects and interactions of antipsychotics, such as AIWG, and – as importantly – the options available to mitigate them. The options would also need to be realistic, in that they are acceptable and available to the patient when needed.

In addition, people with SMI are a vulnerable group. They may be less able to attend to their own needs owing to their illness, for reasons previously described (e.g. negative symptoms, insight loss, being likelier to reside in a socially deprived environment). Consequently, psychiatrists may be duty-bound to do more for them than a clinician might otherwise do for patients without SMI, in the interests of justice. This is so that SMI patients are provided more equal access to healthcare proportionate to their needs and are not unfairly deprived because of their illness.

### Medico-legal implications

The above issues present potential liability issues for psychiatrists. A person can be found negligent if it is proven that they had a duty of care, that they failed to carry this out to an acceptable standard of care and that this led to causal harm to the aggrieved, particularly when harm is foreseeable yet avoidable. The technical nature of medical negligence means expert opinion is sought to ascertain whether a clinician's practice met the acceptable standard of care.^39^ Where opinions vary, the judge need not accept all opinions as valid.^40^ Moreover, the clinician's actions need not be the sole or dominant cause for them to be found negligent.^41,42^ Examples of potentially negligent care include medication side-effects (e.g. tardive dyskinesia from receiving antipsychotics)^43^ and failure to adequately monitor physical effects of antipsychotics.^44,45^ Ultimately, whether a psychiatrist is negligent is decided on the specifics of each case. Nonetheless, one should ideally try to maintain one's routine practice to be well within acceptable standards of care and not require judicial review – even if ultimately not found liable – for negligence.

Conflicting recommendations between psychiatric and non-psychiatric guidelines create ambiguity around the most appropriate treatment for patients with AIWG or obesity in SMI. It becomes unclear whether off-label medications (particularly metformin) should be used (per psychiatric guidelines) or avoided altogether (per cardiology, endocrinology and national body guidelines). It could be argued that psychiatrists have expertise in managing psychotropics and the needs of people with SMI but lack expertise in managing obesity. Conversely, the reverse argument could be made for endocrinologists and cardiologists. Moreover, the lack of endorsement by metformin's manufacturers outside treatment for T2DM could further call into question its suitability as a pure WLM for nondiabetics.

Such ambiguity could cause indecision, hesitation by clinicians in initiating treatment and inaction. Where a decision is made, clinicians would need to decide whose expertise and recommendations take precedence for each patient on a case-by-case basis. Decisions made under these conditions could be seen as ‘controversial’ and potentially more contestable for not meeting the ‘prevailing standard of care’.^46^ This leaves the supervising psychiatrist on shakier ground in terms of liability, especially where compulsory treatment or non-consenting patients are involved. It would thus be preferable if treatment decisions were triangulated between subject expertise, consensus between guidelines and use of medications within their market authorisation.

## Possible interventions

### Compulsory treatment for weight management – an underutilised tool?

Although not ideal, compulsory treatment is recognised as occasionally necessary in psychiatry to maintain patients’ health and safety. In England and Wales, ‘treatment’ under the MHA1983 can include medications, therapy, rehabilitation and nursing care.^47^ It can also extend beyond direct treatment of the SMI and include ‘ancillary’ care for the consequences of SMI and/or as a prerequisite to the core treatment administered,^48^ which has been interpreted as allowing compulsory treatment for psychotropic side-effects under the MHA1983.

However, treatment for AIWG is not typically given in this way. Instead, common practice has limited the practice to side-effects that could only be caused by the medications (e.g. extrapyramidal side-effects of antipsychotics, hypersalivation due to clozapine) and not more common conditions that may be multifactorial or have other known causes (e.g. obesity, constipation). The reasons provided for this distinction were practicality and to avoid the list of medications psychiatrists are expected to certify for use under the MHA1983 being ‘taken to absurd lengths’.^49^

It should however be argued that clinical practice ought to be guided less by clinician convenience and more by patient safety. Indeed, safety – rather than the desire to keep the number of potential applicable treatments as low as possible – would better meet the ‘necessary’ criterion stipulated in *B v Croydon Health Authority* [1994]^48^ around providing ancillary care under the MHA1983. Logic would dictate that treating mild hypersalivation due to clozapine is less ‘necessary’ than treating AIWG in a patient already at high risk of heart disease and/or stroke. If compulsory treatment under the MHA1983 could not be used for medication side-effects (e.g. AIWG) or to prevent cardiometabolic risk factors as complications of SMI, patients would be able to circumvent compulsory treatment for SMI by making it unsafe to remain on treatment and by refusing ancillary care for the core treatment's side-effects.

Furthermore, requiring the undesirable effects of a psychotropic to be the sole possible cause of a condition – as recommended by Kinton et al – before it can be treated as a medication side-effect calls for a higher standard of proof than legally required. The MHA1983 asks that an issue be proven on the ‘balance of probabilities’.^50^ Therefore, a side-effect should be treatable if it is ‘more likely than not’ to be caused by the psychotropic rather than needing it to be proven ‘beyond reasonable doubt’, which is the standard used in criminal trials.

This is not to suggest that all psychiatric patients should routinely receive compulsory weight management. Just because one can does not mean one must or should. Careful consideration would need to first take place in balancing the principles of beneficence, maleficence and justice against autonomy so as to not be needlessly paternalistic. Its use should thus be limited only to patients requiring it, much the way compulsory treatment is used in other aspects of psychiatry.

Examples would include patients who are unable to consent owing to the SMI. Another group would be patients who wish to remain on an obesogenic antipsychotic that they respond well to but whose risk factors might otherwise require it to be changed. Here, rather than restrict freedom, compulsory weight management can potentially support patient autonomy by facilitating their preferred antipsychotic and/or treatment. This could be done by including compliance with a weight management programme as a condition on their community treatment order to motivate them to receive treatment for AIWG. Moreover, including weight management as part of SMI treatment has the potential benefit that patients could receive funding for those treatments if subject to section 117 (after-care) of the MHA1983. This places increased funding responsibility on local authorities, potentially improving patients’ access to weight management treatment.

### Semaglutide injections – treatment of the future?

In February 2022, the National Institute of Social and Care Excellence (NICE) began drafting guidance around treating obesity with semaglutide, a glucagon-like peptide-1 (GLP-1) receptor agonist licensed for this use.^51^ It is administered subcutaneously once weekly. Another GLP-1 receptor agonist is liraglutide, which is also licensed as a WLM. Others, such as exenatide, lixisenatide, albiglutide and tirzepatide, are licensed only to treat T2DM and not obesity.

Owing to its recent introduction, semaglutide use in AIWG has yet to be investigated,^30^ though data on other GLP-1 receptor agonists in this population^30,52^ and on semaglutide in the general population^53^ are promising. The lack of SMI-specific research is unfortunate, as several attributes of semaglutide make it especially useful in this population. Patients with psychosis may find regular injections more acceptable compared with the general population, as injectable long-acting antipsychotics (e.g. aripiprazole, paliperidone, risperidone) are seen as a normal part of psychiatry. Compared with liraglutide (the only other GLP-1 receptor agonist licensed for weight loss), which requires daily injections, the weekly dosage of semaglutide may be more convenient for patients who struggle with adherence and routine. It also integrates well into existing service structures such as weekly care coordinator contact or depot clinics, which then helps facilitate monitoring of treatment adherence.

However, semaglutide may not be suitable for all patients. For instance, the manufacturer advises avoiding use during pregnancy (owing to reproductive toxicity in animal studies) or while breastfeeding (as it has been found to be secreted in breastmilk in animal studies).^54,55^ Similarly, semaglutide can delay gastric emptying, which may make it less desirable among patients already at risk of constipation (e.g. patients treated with clozapine).^54,55^ The proposed NICE guideline also only recommends continuous semaglutide use for up to 2 years.^51^ Although this may be enough to treat AIWG, further support after treatment completion would be beneficial to prevent AIWG from recurring and/or to monitor the need for further courses of treatment in the future.

Introducing semaglutide into psychiatric care presents an opportunity to better integrate local authority, acute hospital and mental health services in patients’ weight management. Existing resources could be pooled for more effective utilisation. For instance, for those unable to self-manage their treatment, care coordinators and/or depot clinics would probably be able to administer semaglutide to patients – a service endocrinologists or acute hospitals might be otherwise unable to independently provide – without a drastic change to their existing workload and routines. Likewise, psychiatrists’ involvement could grant additional legal frameworks (e.g. compulsory treatment) that endocrinologists would not normally have.

Joint working with endocrinologists can provide mental health services with greater expertise and safer prescribing around weight management. This could include better review of previous treatments or ensuring that conservative interventions (e.g. diet, exercise, lifestyle interventions) have been optimised, per the therapeutic indication for semaglutide. As it forms part of psychiatric treatment, costs could also be shared with local authorities (e.g. through section 117 (after-care) of the MHA1983). This may in turn prompt other, less-costly interventions (e.g. gym access, healthy food options) to be made available as part of cost management, which would once again benefit patients by increasing access to such services.

This arrangement has additional liability benefits. Having endocrinologists and psychiatrists working together to manage AIWG would mean that appropriate expertise would be present from both the psychiatric and endocrinological perspectives. It would also provide interdisciplinary learning opportunities to improve and better meet the previously identified training gap among psychiatrists around managing obesity. Likewise, updating psychiatric practice and guidelines to support semaglutide would help resolve the conflict between psychiatric and non-psychiatric recommendations around off-label prescribing for weight management. However, owing to the lack of direct evidence regarding its use, this would likely be difficult to accomplish at present. Further research on semaglutide use in AIWG and obesity in SMI first needs to take place.

## Conclusions

AIWG and obesity in SMI present a problem to patients and clinicians alike, complicated by questions of clinical practice, patient adherence and medico-legal issues. Clearly, psychiatrists – as the principal clinicians in charge of the care of patients with SMI – can and should be doing more, particularly where compulsory treatment is concerned. This article puts forward ideas around what can be done, including changes in practice that can be implemented today, as well as identifying potential areas for further development.

## About the authors

**Kenn Lee**, MBBCh, MRCPsych, is a specialty registrar at Pennine Care NHS Foundation Trust, UK; **Adeola Akinola**, MBChB, MRCPsych, PGDip, LLM, FHEA, is a consultant psychiatrist with Pennine Care NHS Foundation Trust and a lecturer in ethics and law at the University of Manchester, UK; **Seri Abraham**, MBBS, MRCPsych, MSc, is a consultant psychiatrist with Pennine Care NHS Foundation Trust and honorary senior lecturer at Manchester Metropolitan University, UK
